# Dexmedetomidine: the first new kid on the block for preventing cardiac surgery-associated acute kidney injury?

**DOI:** 10.1186/s13054-018-2072-3

**Published:** 2018-06-05

**Authors:** Patrick M. Honore, David De Bels, Thierry Preseau, Herbert D. Spapen

**Affiliations:** 1 ICU Department, Centre Hospitalier Universitaire Brugmann, Place Van Gehuchtenplein,4, 1020 Brussels, Belgium; 2 Emergency Department, Centre Hospitalier Universitaire Brugmann, Brussels, Belgium; 3 Universitair Ziekenhuis Brussel, VUB University, Brussels, Belgium

Cardiac surgery-associated acute kidney injury (CSA-AKI) affects up to 30% of patients undergoing cardiopulmonary bypass (CPB) surgery and is the second most common cause of AKI in the intensive care unit [1, 2]. To date, scarce evidence supports specific measures to prevent CSA-AKI. A recent meta-analysis by Shi and Tie [1] highlights the use of the selective α2-adrenoreceptor agonist dexmedetomidine (DEX) for prevention of CSA-AKI. The authors assume that this renoprotective effect is multifactorial, including both direct (by increasing renal blood flow (RBF) and diuresis) and indirect (by decreasing oxidative and inflammatory “stress”) effects on the kidney [1].

The work of Shi and Tie must be interpreted with caution. Many cardiac surgery patients have pre-existent renal dysfunction or specific comorbidities that enhance the risk to develop AKI. Patients are also exposed to a compilation of per- and postoperative renal “aggressors”, including the CPB procedure itself, aortic cross-clamping time, (poly)transfusion, vasopressor and inotropic treatment, and the use of particular colloid or crystalloid infusions. Also, long-standing venous congestion as a result of increased right ventricular afterload makes the kidneys more vulnerable to “congestive failure” in case of peri-operative hemodynamic instability [2]. Any baseline incongruity regarding these variables between DEX and controls may significantly undermine the value of a meta-analysis. We strongly argue against the idea that DEX provides kidney protection by simply improving RBF. An increase of RBF does not change renal tissue oxygenation but rather augments pre-glomerular arteriovenous oxygen shunting. Medullary hypoxic damage is the key responsible for CSA-AKI. Under normal conditions, the outer medulla already suffers borderline hypoxia and thus becomes particularly sensitive to prolonged or intermittent episodes of low oxygen supply caused by CPB-induced renal vasoconstriction and hemodilution [3]. Moreover, on- and off-pump procedures cause capillary fall-out and capillary flow cessation, respectively, which may aggravate medullary hypoxia [4]. Of note is that propofol anesthesia was also found to significantly reduce incidence and severity of AKI in patients undergoing valvular heart surgery with CPB [5].

Taken together, a potential protective role of DEX in CSA-AKI can only be reliably appreciated when type and duration of surgery, patient characteristics and comorbidities, right heart hemodynamics, and peri-operative therapeutic strategies are taken into account. Furthermore, it requires comparison with other relevant sedatives and a more thorough insight into DEX-mediated effects on compartmental RBF distribution and renal microcirculation.

## Abbreviations

CPB: Cardiopulmonary bypass
CSA-AKI: Cardiac surgery-associated acute kidney injury
DEX: Dexmedetomidine
RBF: Renal blood flow
